# A Narrative Review on Polycrystalline Ceramics for Dental Applications and Proposed Update of a Classification System

**DOI:** 10.3390/ma16247541

**Published:** 2023-12-07

**Authors:** Ernesto B. Benalcázar-Jalkh, Edmara T. P. Bergamo, Tiago M. B. Campos, Paulo G. Coelho, Irena Sailer, Satoshi Yamaguchi, Larissa M. M. Alves, Lukasz Witek, Sérgio M. Tebcherani, Estevam A. Bonfante

**Affiliations:** 1 Department of Prosthodontics and Periodontology, Bauru School of Dentistry, University of São Paulo, Bauru 17012-901, SP, Brazil; 2 Biomaterials Division, NYU Dentistry, New York, NY 10010, USA; 3 Department of Prosthodontics, NYU Dentistry, New York, NY 10010, USA; 4 DeWitt Daughtry Family Department of Surgery, Division of Plastic Surgery, Miller School of Medicine, University of Miami, Miami, FL 33136, USA; 5 Department of Biochemistry and Molecular Biology, Miller School of Medicine, University of Miami, Miami, FL 33136, USA; 6 Division of Fixed Prosthodontics and Biomaterials, University Clinics of Dental Medicine, University of Geneva, 1211 Geneva, Switzerland; 7 Department of Dental Biomaterials, Osaka University Graduate School of Dentistry, Suita 565-0871, Osaka, Japan; yamaguchi.satoshi.dent@osaka-u.ac.jp; 8 Department of Biomedical Engineering, NYU Tandon School of Engineering, New York University, Brooklyn, NY 11201, USA; 9 Hansjörg Wyss Department of Plastic Surgery, NYU Grossman School of Medicine, New York, NY 10017, USA; 10 Department of Production Engineering, Federal University of Technology—Paraná, Av. Monteiro Lobato Km 04, Ponta Grossa 84016-210, PR, Brazil; sergiomt@uepg.br

**Keywords:** zirconia, alumina, restorative ceramics, prosthodontics, fixed dental prostheses

## Abstract

Dental zirconias have been broadly utilized in dentistry due to their high mechanical properties and biocompatibility. Although initially introduced in dentistry as an infrastructure material, the high rate of technical complications related to veneered porcelain has led to significant efforts to improve the optical properties of dental zirconias, allowing for its monolithic indication. Modifications in the composition, processing methods/parameters, and the increase in the yttrium content and cubic phase have been presented as viable options to improve zirconias’ translucency. However, concerns regarding the hydrothermal stability of partially stabilized zirconia and the trade-off observed between optical and mechanical properties resulting from the increased cubic content remain issues of concern. While the significant developments in polycrystalline ceramics have led to a wide diversity of zirconia materials with different compositions, properties, and clinical indications, the implementation of strong, esthetic, and sufficiently stable materials for long-span fixed dental prostheses has not been completely achieved. Alternatives, including advanced polycrystalline composites, functionally graded structures, and nanosized zirconia, have been proposed as promising pathways to obtain high-strength, hydrothermally stable biomaterials. Considering the evolution of zirconia ceramics in dentistry, this manuscript aims to present a critical perspective as well as an update to previous classifications of dental restorative ceramics, focusing on polycrystalline ceramics, their properties, indications, and performance.

## 1. Introduction and Background

Zirconia polymorphism has been widely applied in dentistry and orthopedics in a variety of surgical and reconstructive scenarios [1]. The outstanding mechanical properties and biocompatibility of the first-introduced zirconia, as well as its white coloration and opacity, lead to its broad application as an alternative to metallic frameworks to improve esthetics in both teeth- and implant-supported single and multiple units fixed dental prostheses (FDPs) [2]. While veneered zirconia presents a high success rate for dental- and implant-supported single crowns, its clinical performance as a framework for FDPs has revealed a high rate of mechanical complications, where cohesive fractures of the veneered porcelain have been the main finding [3,4]. To eliminate the possibility of chipping, the indication of zirconia in its monolithic form led to significant efforts to improve the esthetic appearance of the material. Since then, optically improved zirconias have been produced through different modifications to the composition and processing methods, aiming to minimize light scattering within the material [5,6]. Considering the difficulty of balancing optical and mechanical properties in dental materials science [7], and the complexity of zirconia’s metastability, the quest for a monolithic material that is resistant enough to manufacture long-span FDP, while presenting favorable optical properties to achieve esthetic results in its monolithic form and that is completely stable in the oral environment, remains a substantial challenge. 

The literature regarding the clinical performance of all-ceramic materials evidenced that no ceramic system has reached the success rate of gold standard metal ceramics for dental- [3] and implant-supported FDPs [8]. Furthermore, strong clinical evidence has suggested that zirconia should not be considered as a first-choice material for the manufacture of implant-supported FDPs due to the high risk of mechanical complications [9,10]. As an alternative, novel zirconia materials and polycrystalline ceramic composites have been developed as promising alternatives to the previous generations of dental zirconias [11,12,13]. Therefore, this manuscript aims to present a narrative review on zirconia and polycrystalline ceramic composites, as well as a critical perspective on their current clinical performance and ongoing developments. The authors also present an update to previous classifications of restorative ceramics [14,15] to facilitate communication, focusing on the evolution of polycrystalline ceramic materials for the manufacture of long-span FDPs.

## 2. Polycrystalline Dental Ceramics 

From a materials science perspective, polycrystalline materials are solids comprising small crystals, also known as grains, that are separated by grain boundaries and present a random crystallographic orientation [16]. In general terms, polycrystalline ceramics are nonmetallic inorganic ceramic materials that do not contain a glass phase, providing a material with higher strength and fracture toughness relative to glass ceramics [14].

**Figure 1:**
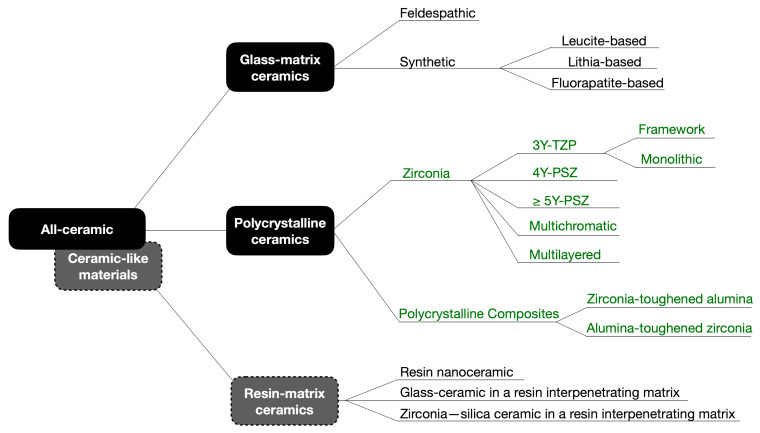
Schematic diagram of the updated classification of dental ceramics and ceramic-like materials, adapted from Gracis et al. (2015) [14] (the updated sections are presented in dark green). As glass-infiltrated ceramics produced by slip-casting and pure alumina ceramics have been withdrawn from the market, these groups were not considered in this update. Ceramic-like materials, such as resin-matrix ceramics represented by resin composite blocks for CAD/CAM, currently have more compositions than those presented in this paper. Due to the prolific launching in the market of new such products, including 3D-printed materials, they are not explored in this review. Modified with permission from CopyRightClearanceCenter.

The classification of restorative ceramics according to their composition proposed by Gracis et al. has been used in the dental literature since its publication in 2015 [14]. Considering the evolution of polycrystalline ceramics in recent years, an update to this classification seems necessary and is presented in Figure 1. Following the logic of the original proposal, zirconia materials have been classified according to its composition and clinical applications. Additionally, as glass-infiltrated ceramics produced by slip-casting and pure alumina ceramics have been withdrawn from the market, both groups have been excluded in this update. Ceramic-like materials, such as resin-matrix ceramics found in resin composite blocks for CAD/CAM, offer a wider range of compositions than those depicted in Figure 1. These materials, indicated for final restorations, are not only available for milling but most recently for 3D printing. Due to the prolific launching in the market of new such products, they are not explored in this review. Since the scope of this review are ceramic materials for long-span FDPs, the updates on zirconia and polycrystalline materials are presented in the following section. 

### 2.1. Properties and Classification of Dental Zirconias

Composition, processing, and microstructure are critical factors that rule the overall properties and performance of ceramic materials, and dental zirconias are no exception [17]. Due to its allotropic behavior, pure zirconia presents three distinct temperature-dependent crystalline structures: (1) monoclinic, stable at room temperature up to 1170 °C; (2) tetragonal, stable up to 2370 °C; and (3) cubic, stable from 2370 °C up to the melting point [1]. Interestingly, each crystalline structure presents not only a different atomic arrangement, but also different optical and mechanical behaviors. Such behaviors have been used to tailor restorative materials with different properties that have been recommended for a diversity of clinical scenarios [15].

#### 2.1.1. 3Y-TZP

**Figure 2:**
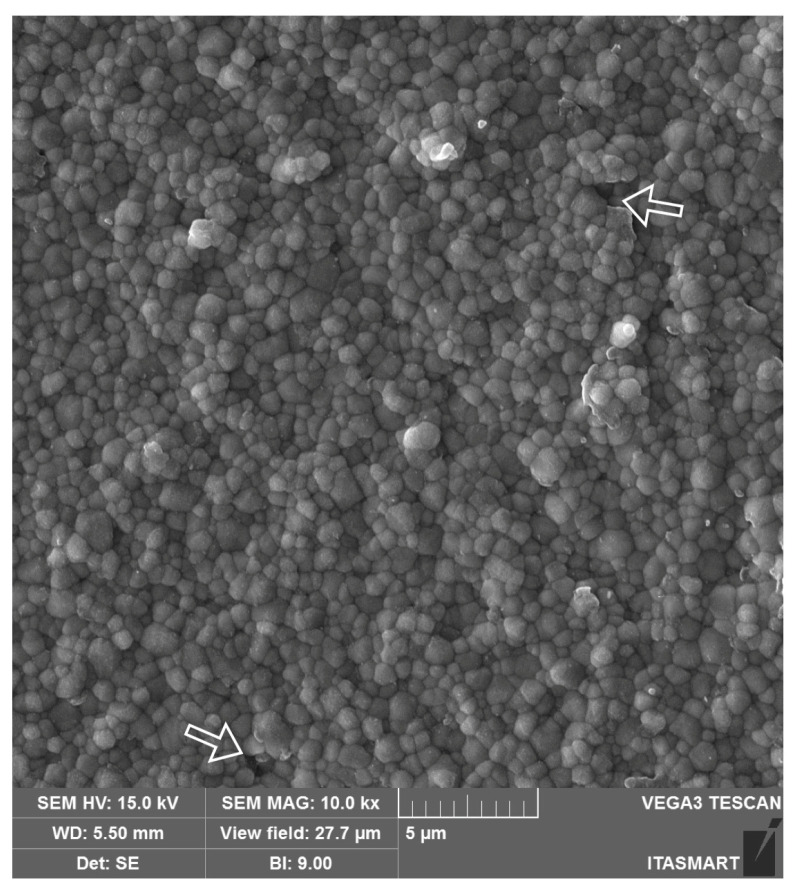
Scanning electron microscope (SEM) image of the 3Y-TZP framework. A dense microstructure is depicted with regular and homogeneous spherical zirconia grains and few microstructural defects (white arrows), which may be related to ceramic processing.

Yttrium-stabilized tetragonal zirconia polycrystals (Y-TZPs), the first zirconia introduced in dentistry, are chiefly composed of the tetragonal phase and are stabilized at room temperature with the addition of 3 mol% of yttrium oxide (3Y-TZP). The use of 3Y-TZP for frameworks, also known as the first generation of dental zirconias, was introduced as an alternative to metal ceramics for esthetic dental treatments because of its high mechanical properties and white coloration [2,15]. The partial stabilization of tetragonal zirconia with 3 mol% of yttria and a small amount of alumina (0.25 wt%) provides the material with a dense microstructure (Figure 2) and high fracture toughness due to crack compression through transformation toughening [18]. This mechanism consists in the transformation of the grains around the crack tip from the tetragonal to the monoclinic phase (t-m) when the material is submitted to stress and is followed by a volumetric increase in grains (3–5%). Such transformation results in the phenomenon known as R-curve behavior, where the volumetric change in the transformed grains create beneficial compressive stresses that hinder crack propagation and ultimately increase the material’s fracture toughness [1], granting 3Y-TZP the highest mechanical properties among all ceramic systems marketed in dentistry [2]. 

Although stress-mediated transformation is the reason for zirconia’s mechanical performance, it also makes it susceptible to a steady and continued transformation from the tetragonal to the monoclinic (t-m) phase due to a combination of stress and humid environmental exposure, also known as low-temperature degradation (LTD) [19,20]. This phenomenon has been reported as starting at the surface and propagating within the material through a “nucleation and growth” mechanism, with a detrimental effect on the mechanical properties due to stress accumulation and the appearance of micro-cracks [21]. Furthermore, the transformation might be accompanied by grain growth, grain extrusions, and grain pull-out, along with microstructural defects, further compromising the mechanical behavior and surface roughness of the material over time [22].

Along with high mechanical properties, the opacity of first-generation dental zirconias, due to the natural birefringence of the tetragonal phase, has led to its clinical application mainly as framework material to be covered by a feldspathic porcelain to achieve adequate esthetic results [23]. Moreover, even when indicated for bilayered reconstructions, the veneering process has been shown to trigger t-m phase transformation at the porcelain/3Y-TZP interface due to moisture from the porcelain slurry and its heating in the furnace [24]. This transformation in the interface has been suggested to increase residual stress within the veneered porcelain, which might compromise the clinical performance of the restorative treatment [25]. Furthermore, clinical studies have consistently reported the cohesive fracture of the veneered porcelain as a recurrent complication in bilayered zirconia systems, especially in long-span FDPs with zirconia frameworks [3,9,26]. Such findings, attributed to the significant differences in the coefficient of thermal expansion (CTE) between the zirconia and the veneering porcelain [27] and the reduced fracture toughness of the porcelain that is leucite-free [28], have led to the development of zirconias with improved optical properties to be used as monolithic restorative materials.

#### 2.1.2. From Porcelain-Fused to Zirconias (PFZs) to Monolithic Zirconias

**Figure 3:**
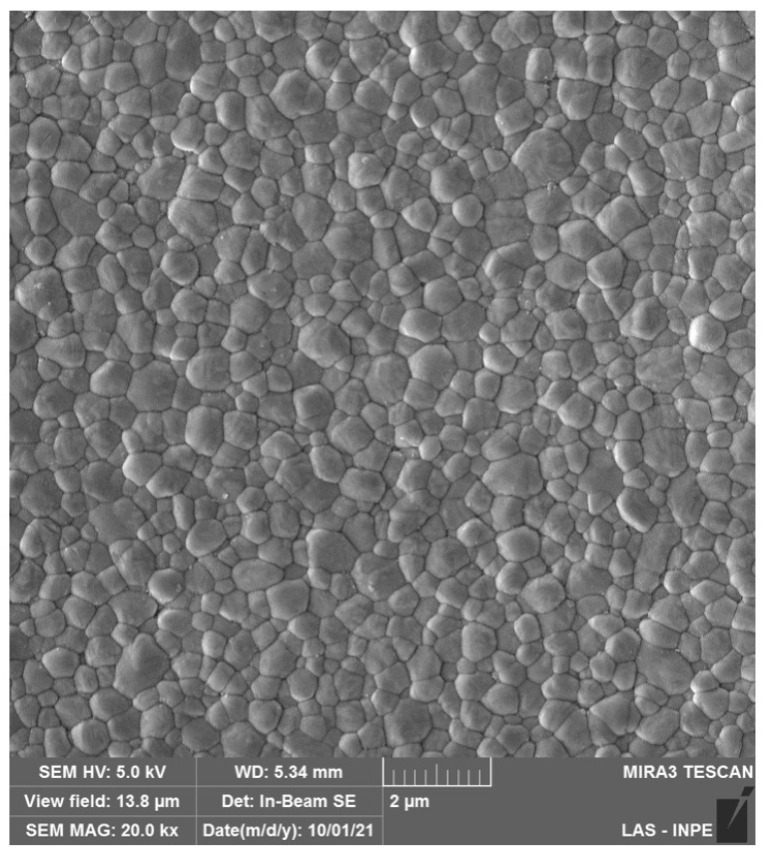
SEM micrograph of monolithic 3Y-TZPs, where a dense surface with slightly larger grains and fewer structural defects are observed in comparison to 3Y-TZPs of the first generation, being used as a framework material.

Monolithic 3Y-TZP was developed by significantly reducing the Al_2_O_3_ (≦0.1 wt%) content and by eliminating porosity through sintering at higher temperatures [15], improving zirconia’s optical properties (microstructure is depicted in Figure 3). However, the indication of monolithic 3Y-TZP, also referred to as second-generation zirconia, for the manufacture of prostheses without porcelain veneering resulted in a larger exposure of zirconia to the oral environment, which raised concerns about the impacts of LTD on dental prostheses [29]. In addition to extensive laboratory work corroborating controlled LTD by means of artificial hydrothermal aging and the effects on zirconia microstructure and mechanical properties, the literature regarding LTD of 3Y-TZP has evidenced that, after only 60 to 100 days in situ, 3Y-TZP presented a significant increase in the surface roughness, flexural strength, and phase transformation in the oral environment [30]. Furthermore, the reduction in the alumina content in monolithic 3Y-TZPs (≦0.1 wt%) led to a higher susceptibility of the LTD effects [29], where t-m transformation has been shown to be almost threefold higher than first-generation 3Y-TZP, resulting in altered optical and mechanical properties [31,32].

Although monolithic 3Y-TZPs present improvements in translucency when compared to infrastructure 3Y-TZP, their optical properties do not reach the esthetic levels that would make them competitive to any glass-matrix ceramics, especially to be used in the anterior area. While extrinsic pigments were introduced in an attempt to optically mimic dental structures [33], the esthetic results achieved with monolithic second-generation zirconia were limited due to the natural opacity of the birefringent tetragonal crystals and the subsequent light scattering within the materials’ structure.

#### 2.1.3. 4Y-PSZ

**Figure 4:**
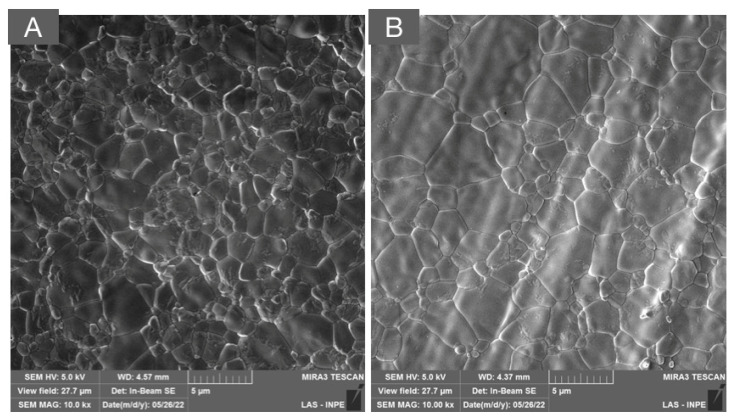
SEM micrographs of (**A**) 4Y-PSZ and (**B**) 5Y-PSZ depicting a dense microstructure with a predominance of cubic grains that are significantly larger than those of the 3Y-TZP versions used as framework and monolithic, as presented in Figure 2 and Figure 3, respectively. The high content of the non-birefringent cubic phase allows for a higher light transmittance and, therefore, higher translucency.

In the effort to further improve translucency, subsequent generations of dental zirconia were introduced by the addition of transparent, non-birefringent, cubic-phase stabilization with higher amounts of yttrium oxide (5Y first and then 4Y) [6]. The stabilization of approximately 25% of the cubic phase achieved with 4 mol% of yttrium oxide produced a partially stabilized zirconia with increased translucency in relation to 3Y-TZP for monolithic applications. The microstructure of 4Y-PSZ is depicted in Figure 4A, where larger cubic grains are clearly observed compared to 3Y-TZP. 

While improved translucency was achieved with this approach, cubic zirconia does not undergo stress-induced transformation, which leads to a slight reduction in the mechanical properties along with its range of application. With a flexural strength ranging from 600 to 1000 MPa and a fracture toughness from 2.5 to 3.5 MPa·m^1/2^ [34], 4Y-PSZ ceramics have been recommended for the manufacture of monolithic single crowns and three-unit fixed dental prostheses in the anterior and posterior regions. However, its clinical performance in the mid- and long term has not been reported in clinical trials [15].

#### 2.1.4. ≥5Y-PSZ

The progressive increase in yttrium oxide (5–8% mol) in zirconia materials, and the subsequent stabilization of higher amounts of the non-birefringent cubic phase, led to the development of the so-called “ultra-translucent zirconias” (the microstructure is depicted in Figure 4B). Although a significant increase in translucency was observed compared to other monolithic zirconias, the stabilization of over 50% of the cubic phase led to a notable reduction in the flexural strength (400–900 MPa) and fracture toughness (2.2 to 2.7 MPa·m^1/2^) compared to 3Y-TZP used as frameworks (1200–1500 MPa; 3.5–4.5 MPa·m^1/2^), to monolithic 3Y-TZPs (900–1300 MPa; 3.5–4.5 MPa·m^1/2^), and to monolithic 4Y-PSZ (600–1000 MPa; 2.5 to 3.5 MPa·m^1/2^) [15]. Therefore, “ultra-translucent zirconias” have been recommended for partial/total single-unit reconstructions and some of them for short-span fixed dental prostheses in the anterior region [15]. However, clinical evidence regarding the mid- and long-term performance of these materials is yet to be reported.

Due to their high yttrium and cubic-phase content, 4Y- and 5Y-PSZ have been commonly grouped within the same “ultra-translucent” family. However, laboratory studies have indicated different mechanical, optical, and aging behaviors for 4Y- and 5Y-PSZ [35,36,37]. These differences may be explained due to a higher phase stabilization with 5 mol% of yttrium oxide, which produce over twice the percentage of cubic phase stabilization (50%) compared to 4 mol% (25%). Such differences in composition and microstructure also grants 4Y-PSZ a lower hydrothermal aging resistance and higher mechanical properties compared to 5Y-PSZ [38].

Furthermore, considering the range of clinical indications of highly translucent zirconias, a pragmatic comparison readily emerges with the commercially available glass-matrix ceramics indicated for similar situations. Among glass ceramics, lithium disilicate and other lithia-based ceramics have been recognized for the favorable balance between optical and mechanical properties and are frequently preferred over zirconias for the manufacture of single-unit partial and total reconstructions [7]. Along with the higher translucency provided by its high glass content [39], silica ceramics’ acid etching, silanization, and adhesion are straightforward and well-documented procedures that have been shown to provide long-lasting bonding and outstanding survival rates in the long term [40,41]. The substantial difference in clinical evidence regarding the performance of both materials make it difficult to justify the selection of third-generation dental zirconias over lithium disilicate, which presents more predictable esthetic outcomes [17,39]. 

#### 2.1.5. Multichromatic Zirconia

Multichromatic zirconia was introduced in dentistry through the addition of layers with different pigments to provide a graded shade between the cervical and the incisal portion of the restoration to mimic the natural tooth appearance [11]. The first system comprising layers with different pigmentations (Katana, Kuraray, and Japan) presented three different zirconia compositions: (1) ML: multi-layered zirconia composed of 3Y-TZP; (2) STML: super-translucent multi-layered zirconia composed of 4Y-PSZ; and (3) UTML: ultra-translucent multi-layered zirconia composed of 5Y-PSZ.

Ironically, the microstructural analysis of multichromatic systems, commercially called “multilayer” by some companies, revealed the same yttrium content and cubic fractions in the different layers of each material. Pigment compositions were the only difference among the layers, which, as expected, led to significant differences in shade, but not in the translucency of the layers [11]. Additionally, while the graded shade obtained with multichromatic zirconias resulted in a more esthetic option regarding previous monolithic zirconias, the microstructural features as well as the fundamental disadvantages of each type of zirconia remains a concern.

#### 2.1.6. Multilayered Zirconia

**Figure 5:**
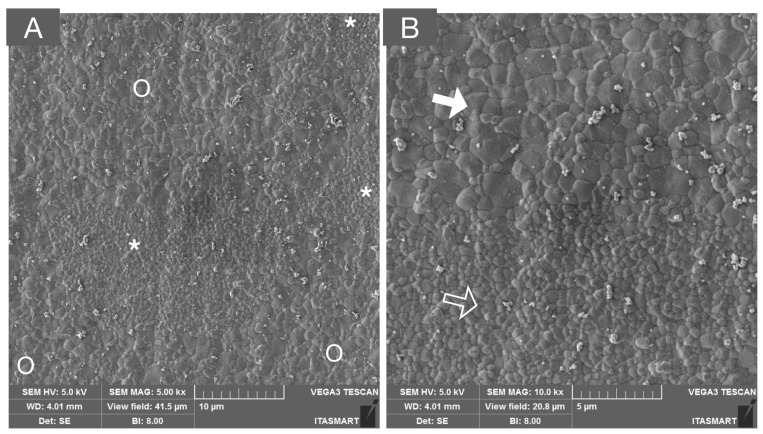
SEM micrograph of a multilayered system composed of 3Y-TZP and 5Y-PSZ, depicting the transition layer composed of interpenetrated areas of tetragonal (white asterisks and unfilled arrow) and cubic grains (white circles and solid white arrows) in low (**A**) and high (**B**) magnifications.

The trade-off between translucency and strength observed with the increased amount of the cubic phase encouraged a different approach to provide resistant and esthetic materials for monolithic applications [17]. Materials provided with a layered structure composed of different compositions of dental zirconias have been suggested to provide graded structures that mimic not only the shade, but the translucency and esthetic appearance of natural teeth. The microstructural characterization of such materials has evidenced a gradient on the yttria content from the gingival to the incisal regions, along with a progressive increase in the content of the cubic phase and, therefore, translucency [42]. A representative micrograph of a multilayered system composed of 3Y-TZP and 5Y-PSZ is presented in Figure 5, where the transition layer composed of the interpenetrated areas of tetragonal and cubic grains can be observed.

The association of different zirconia generations within the same material had a significant impact in the esthetic outcome of zirconia monolithic restorations. Laboratorial characterizations suggest that commercially available multilayered zirconia blanks differ predominantly in the intermediate layers. Therefore, the milling position in the blocks should be carefully evaluated for each individual clinical case when using multilayer zirconia as the restorative material [43]. While an association of the beneficial aspects of each zirconia family is expected in multilayered systems, it has been suggested that the fracture resistance of yttria-graded multilayered systems is determined by the amount of the weaker zirconia phase at the occlusal portion of the restoration rather than enforced by the stronger zirconia at the cervical part of the crown [44].

Furthermore, the presence of metastable 3Y-TZP, which bestows high mechanical properties to the ensembled material, also makes it susceptible to low-temperature degradation. The kinetics of hydrothermal aging in multilayered systems suggest that t-m phase transformation remains a concern in the 3Y-TZP layer, where pronounced phase transformation has been reported after artificial aging [45,46]. Otherwise, the layers with a higher amount of yttria and cubic phase demonstrated minimal to non-phase transformation when submitted to the same hydrothermal aging protocols [46].

### 2.2. Clinical Performance of Dental Zirconias for FDPs

Considering the evolution of dental zirconia and the modifications to its composition and microstructure, it is of utmost importance to correlate the impact of such modifications with its clinical performance and potential complications in both dental- and implant-supported FDPs.

Regarding the clinical performance as dental-supported single crowns, it is noteworthy that porcelain-veneered zirconia crowns have shown promising success rates after 5 and 10 years (94% and 90%, respectively) [4,47,48]. However, the performance of porcelain-veneered zirconia FDPs demonstrated significantly lower survival rates when compared to metal ceramics after 10 years in function [49].

The primary technical complication reported for porcelain fused to 3Y-TZP framework reconstructions is the chipping of the veneer material (up to 18% after 10 years) [48,50]. This technical complication has been frequently observed in implant-supported prostheses and reported in the recent European Academy for Osseointegration (EAO) consensus, which showed that porcelain veneer fracture rates of approximately 22.8% in partial and 34.8% in full-arch implant-supported FDPs within 5 years seem clinically unacceptable [9,10]. Therefore, the development of monolithic zirconias described in the previous section was considered as a promising alternative to reduce clinical complications.

To date, however, there is a lack of scientific information regarding survival rates on the medium- and long-term performance of monolithic zirconia FDPs [8]. For single crowns, the short-term clinical data (1-year follow-up) has suggested similar survival rates for monolithic 3Y-TZP regarding metal ceramics. Nevertheless, the esthetic assessment relative to natural adjacent dentition was inferior for monolithic zirconia crowns [51]. Partially veneered monolithic 3Y-TZP has been suggested as an alternative to achieve esthetic results and to avoid the chipping of the veneered porcelain in functional areas submitted to occlusal stress. However, a clinical study with 3 years of follow-up evidenced that chipping remained a concern [52]. The evaluation in up to 5 years for 39,827 zirconia prostheses reported a failure rate of 0.71% for crowns and 2.60% for PPFs, suggesting high survival rates for monolithic second-generation zirconias in the short term [53]. Furthermore, a systematic review that evaluated the effect of prosthetic material and design on the clinical outcomes of implant-supported FDPs in the posterior area with a mean follow-up of 3 years revealed that monolithic and partially veneered zirconia exhibit lower ceramic fracture and chipping rates (0.18%) compared with porcelain fused to metal (2.20%) and conventionally veneered 3Y-TZP (4.95%) [54]. Moreover, while clinical evidence regarding full-arch implant supported monolithic and partially veneered zirconia has suggested promising short-term success, long-term data from studies with a strong level of evidence are still lacking [55].

In addition to the lack of long-term clinical validation, the use of monolithic 3Y-TZP has raised concerns regarding its direct exposition to the oral environment and the effects of LTD on the material’s properties and microstructure [29]. The hydrothermal degradation process has been reported to occur as early as after 60 days in vivo and to produce a significant amount of phase transformation associated with increased surface roughness [31]. Furthermore, several laboratorial studies have shown LTD to affect the optical and the mechanical properties of the monolithic 3Y-TZP [31,32,56,57,58]. However, there is a paucity of clinical data regarding LTD in dentistry and only a handful of studies have presented information regarding the hydrothermal degradation of zirconias in vivo [31,59]. Considering the complexity of low-temperature degradation and its repercussion in the orthopedic field, an in-depth explanation and review of laboratorial and clinical data are presented in the following section.

Regarding the clinical performance of ultra-translucent zirconias (≥4Y-PSZ), multichromatic, and multilayered systems, to date, clinical data comprise a small number of case reports [60,61] as well as studies with a reduced number of patients and short-term follow-up periods [62,63,64]. Therefore, well-designed clinical trials are warranted to elucidate the clinical behavior of novel zirconia materials, particularly, regarding the performance of long-span FDPs manufactured with novel multilayered systems.

### 2.3. Low-Temperature Degradation of Dental Zirconias

The LTD of zirconia was first described in the early 1980s as a continued transformation from the tetragonal to the monoclinic phase due to a combination of stress and humid environment exposition [20]. Theories based on the interaction between water molecules and zirconia grains increasing the tension in the material have been proposed as the mechanism by which LTD occurs in 3Y-TZP, but there is no consensus in the literature [65]. This time-dependent process has been suggested to start at the surface and then proceed to the interior of the zirconia by a “nucleation and growth” process, which can lead to grain pull-out, increased surface roughness, and the appearance of microcracks that create a path for water molecule penetration, promoting further phase transformation [21,22,57,66].

The LTD process had worldwide repercussions in 2001, when hundreds of hip replacements failed after 1 to 2 years due to an accelerated LTD process, generating high morbidity scenarios and concerns about the hydrothermal stability of the material [22]. Thereafter, the use of 3Y-TZP as an orthopedic material was largely abandoned in Europe and the United States due to its instability and the risk of increased wear and aseptic loss of the prostheses [67]. These failures led to a paradigm shift in the manufacture of orthopedic zirconia prostheses to avoid such morbid events. Ironically, the episodes of failure of zirconia hip prostheses that had a disastrous effect on orthopedics caused little or no concern in the dentistry field, where zirconia has been broadly used for over 20 years. Such discrepancy between the different areas may be attributed to the lower morbidity resulting from the failure of dental prostheses compared to an orthopedic device and to the lack of knowledge of aging and its impact in the dental field [67].

**Figure 6:**
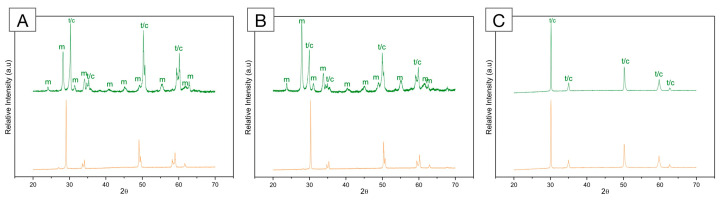
X-ray diffraction (XRD) pattern of (**A**) framework 3Y-TZP; (**B**) monolithic 3Y-TZP; and (**C**) 5Y-PSZ before (orange, bottom) and after (green, top) artificial aging in a hydrothermal reactor for 20 h at 134 °C under 2.2 bars of pression. A significant increase in monoclinic peaks (m) is depicted for both 3Y-TZPs, with higher peaks recorded for the monolithic, second-generation 3Y-TZP. Otherwise, 5Y-PSZ presents characteristic tetragonal (t) and cubic (c) peaks and no significant alterations after aging.

The susceptibility to degradation is dependent on the microstructure and composition of the material. Factors such as density, grain size, as well as quantity, distribution, and type of stabilizers, along with the presence of residual stress, may determine the susceptibility of dental zirconia to phase transformation [66,68]. While framework and monolithic 3Y-TZP have been shown to be susceptible to hydrothermal degradation, zirconias with higher amounts of yttria (≥4Y-PSZ) have demonstrated significant resistance to LTD [69,70,71]. Figure 6 presents the X-ray diffraction (XRD) patterns of framework and monolithic 3Y-TZPs as well as 5Y-PSZ before and after artificial aging in a thermal reactor. The evaluation of these diffractograms demonstrates a significant susceptibility of 3Y-TZP to hydrothermal degradation and no phase transformation in 5Y-PSZ. Such behavior has been observed to remain in the multilayered systems, where the layers containing less yttrium oxide and a reduced cubic phase were susceptible to phase transformation and the layers with higher yttria and cubic phase presented almost no transformation after artificial aging [45,46]. Due to its high yttrium and cubic phase content, it is commonly considered that ≥4Y-PSZ materials do not undergo phase transformation after aging. However, studies with different experimental setups have indicated varying aging behaviors for 4Y- and 5Y-PSZs, where phase transformation has been reported for the former after exposure to mechanical and hydrothermal stimuli in contrast to the latter [36]. This might be explained by the lower phase stabilization with 4 mol% of yttrium oxide, which also grants 4Y-PSZ superior mechanical properties compared to 5Y-PSZ [39].

Remarkably, studies on the hydrothermal stability of the 3Y-TZP used in dentistry have shown considerable variability regarding t-m phase transformation after accelerated artificial aging. Two systematic reviews on LTD of 3Y-TZP zirconias demonstrated a wide variation in the monoclinic phase content after aging, ranging from 2.13% to 81.4% [72] and from 8.7% to 81% [73]. This variability has been correlated in laboratorial studies to significant alterations in the surface roughness and optical and mechanical properties of 3Y-TZP after aging [74,75,76]. Nevertheless, such variations are not merely related to the zirconia’s composition but also to the aging methods and parameters used in different in vitro studies [69]. 

The concerns that were raised after the events of 2001 led to the implementation of ISO 13356 for implantable devices, where an accelerated aging test at 134 °C for 5 h at 2.2 bars was recommended and suggested the presence of up to 25% of monoclinic phase after aging as acceptable [77]. Moreover, a systematic review regarding low-temperature degradation in dental zirconias suggested that increased aging exposition (20 h) is necessary to trigger enough t-m phase transformation to negatively affect the mechanical properties of 3Y-TZP [72]. While autoclave aging has been widely used for in vitro research, the significant variations in pressure and temperature during sterilization cycles are of concern because the sample remains within the desired parameters for a limited time. Considering that the aging kinetics of zirconia can occur faster in the oral environment than in in vitro simulations, extrapolations to in vivo settings should be made with caution [78]. From a critical perspective, it seems reasonable to suggest that the hydrothermal autoclave aging method underestimates the in vivo metastability of 3Y-TZP in the oral environment. As an alternative, the use of hydrothermal reactors for accelerated aging tests has been proposed to trigger a more aggressive phase transformation, where samples remain immersed in water at a constant pressure/temperature during the entire aging process [69]. 

While in vitro studies provide a necessary background to better understand a material’s properties and behavior, the possible correlation between artificial aging and clinical scenarios is limited. The complexity of the in vivo environment with variations in temperature, pH, humidity, and occlusal forces are far from being accurately represented by the in vitro testing of geometric samples. Therefore, clinical studies evaluating ex vivo prostheses are critical to elucidate the mechanisms behind LTD in prosthodontics and to establish correlations between in vitro and in vivo testing, particularly considering the presence of occlusal loads during functional and para-functional activities, where occlusal surfaces have been shown to present significant differences in aging patterns regarding non-loaded surfaces [59].

Within this context, a comprehensive prospective clinical trial with ex vivo monitoring has led to a series of publications. Koenig et al. (2021) evaluated 101 monolithic 3Y-TZP posterior crowns at 6 months post-insertion and then yearly for 5 years. After two years, the t-m phase transformation in axial areas presented conventional nucleation and growth process. However, in occlusal areas, surface crushing and grain pull-out from the clusters was observed as a consequence of associated tribological stress, which may induce an underestimation of the aging process when the evaluation is limited to the quantification of monoclinic phase per se [59]. After 5 years, the authors observed a continued non-uniform propagation of phase transformation in the non-loaded surfaces and tribological stress in the loaded areas, which caused the monoclinic grains to emerge from the clusters [79]. According to the authors, it is likely that LTD initially triggers surface roughness and microcracking and that tribological stresses cause the monoclinic grains to pull away as the transformation progresses. Furthermore, the aforementioned evidence concerning the in vivo effects of LTD on 3Y-TZP systems suggests that aging kinetics can be up to three times faster than the conventionally accepted in vitro–in vivo extrapolations for autoclave aging [78]. While aging-resistant materials are highly desirable in clinical practice to avoid the long-term consequences of hydrothermal instability, the development of comprehensive testing methods that associate aggressive hydrothermal aging and mechanical stresses are required to better reproduce in vitro the effects of hydrothermal aging in vivo. Therefore, comprehensive ex vivo and retrieval analyses of failed 3Y-TZP prostheses are warranted.

Among the several approaches applied to overcome LTD, polycrystalline composites have been proposed, and depending on the predominant phase, these composites are named zirconia-toughened alumina (ZTA) and alumina-toughened Zirconia (ATZ). 

### 2.4. Polycrystalline Composites (ZTA and ATZ) for Dental Applications

**Figure 7:**
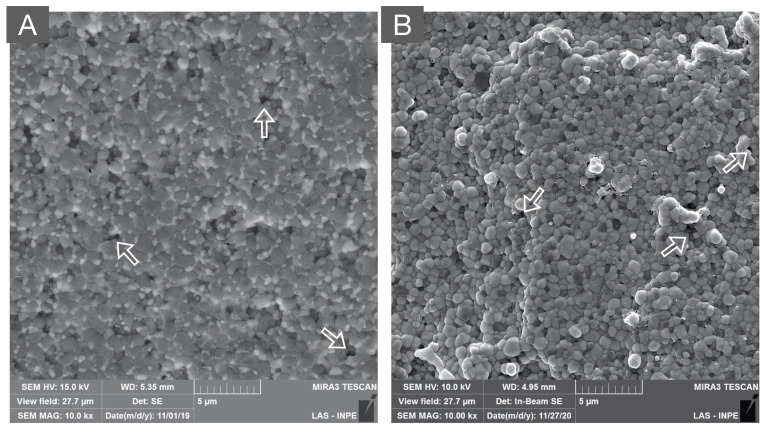
SEM micrograph of: (**A**) zirconia-toughened alumina (80% alumina and 20% zirconia) and (**B**) alumina-toughened zirconia (80% zirconia and 20% alumina) composites. Both polycrystalline composites present a dense microstructure with secondary phases that are homogeneously distributed in the alumina and zirconia matrices, respectively. Moreover, the white arrows indicate microstructural defects originating from ceramic processing.

As an alternative to overcome the stability issues of 3Y-TZP at room temperature, the addition of zirconia particles to an alumina matrix (ZTA) and the addition of alumina particles to a zirconia matrix (ATZ) have been proposed [80,81]. In both scenarios, the addition of a disperse secondary phase has been shown to be efficient in limiting the progression of phase transformation under different laboratorial aging protocols [82,83]. In ZTA composites, this mechanism seems to lie on the limited interconnectivity of zirconia grains by the alumina matrix with an interruption of the nucleation and growth mechanism associated with LTD [84]. Otherwise, the presence of uniformly dispersed alumina particles with a greater elastic modulus in ATZ composites has been suggested to constrain 3Y-TZP grains in the ceramic matrix impeding an extensive t-m transformation [85]. SEM images depicting the microstructures of the experimental ZTA and ATZ are presented in Figure 7A and Figure 7B, respectively.

As LTD propagates through the nucleation of a zirconia grain and the growth and transformation of neighboring grains, the alumina/zirconia ratio dictates the aging resistance as well as the overall properties of the composites [86]. For instance, it has been suggested that the maximum fraction of zirconia in a ZTA composite to limit the propagation of the t-m transformation is related to the interconnectivity of the zirconia grains within the alumina matrix. This fraction, known as the percolation threshold, has been suggested to correspond to 16% of zirconia dispersed in the alumina matrix [87]. However, studies in the dental and orthopedic fields have evidenced interesting results for composites with 15 to 30% of zirconia in their composition [76,88]. In such a proportion, ZTA presented a significant increase in strength in relation to pure alumina, an R-curve behavior suitable for their application in areas of high mechanical demand, and high hydrothermal stability [86,87].

**Figure 8:**
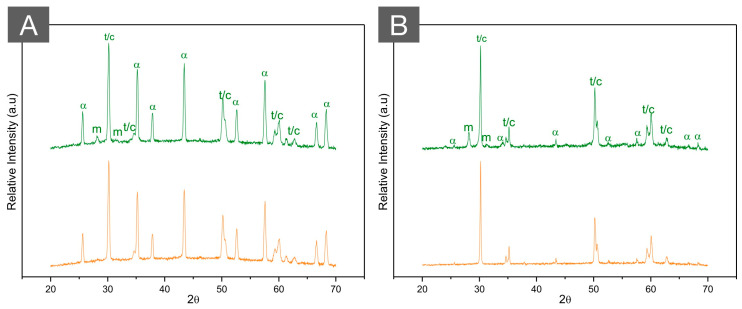
XRD patterns of (**A**) ZTA (70/30) and (**B**) ATZ (80/20) before (green, top) and after (orange, bottom) autoclave aging for 20 h at 134 °C under 2.2 bars of pression. While the high hydrothermal stability of ZTA is observed with almost no modifications in the diffractogram after aging, a limited amount of phase transformation is depicted in ATZ after aging. Letters represent (m) monoclinic peaks, (t) tetragonal peaks, (c) cubic peaks, and (α) alpha alumina peaks.

Recent ZTA formulations synthesized for dental applications with 80/20 and 70/30 alumina/zirconia ratios have shown a promising combination of flexural strength (860 and 900 MPa, respectively) and hydrothermal stability (less than <3% of the m phase) in comparison to framework and monolithic 3Y-TZPs (9% and 26%, respectively) after 20 h of autoclave aging [76,88]. The XRD patterns of an experimental ZTA are presented in Figure 8A, where a limited t-m phase transformation can be observed after aging. Remarkably, the flexural strength, optical properties, and surface roughness parameters of both ZTA compositions have been reported to remain stable after accelerated autoclave aging for 20 h. 3Y-TZP, on the other hand, presented important variations in flexural strength, fatigue behavior, optical properties, and surface roughness parameters after aging [89,90]. Such findings potentially suggest that ZTA is a promising alternative as a framework material for long-span dental prostheses with a great masking ability to offset darkened substrates, such as titanium implant abutments, and endodontically treated teeth, providing a resistant and hydrothermally stable alternative to the 3Y-TZP framework. 

Despite these encouraging findings, the underestimation of the LTD process of zirconia materials mentioned in the previous section is of concern, even for zirconia/alumina composites. While ZTA composites, particularly Biolox Delta (CeramTec, Plochingen, Germany), have been considered as the gold standard for hip replacement in the orthopedic field, several studies have reported that ZTA femoral head prostheses retrieved after short and mid-terms exhibited significant amounts of phase transformation [91,92,93]. For instance, a comparative analysis between ZTA hydrothermally aged for 48 h in an autoclave and ZTA femoral heads retrieved after 2.7 years of function revealed substantial differences in the amount of tetragonal zirconia transformed to its monoclinic phase [94]. In this study, the in vitro autoclave aging of ZTA for 48 h, almost ten times the exposure recommended in ISO 13356, resulted in a monoclinic phase transformation from 12% to 21%. In contrast, the femoral heads retrieved after only 2.7 years of function presented a monoclinic phase concentration of 33%, which supports the concern that previously accepted in vitro to in vivo extrapolations underestimate the amount of phase transformation in vivo [94].

In vivo, femoral head prostheses are subjected to high stress and abrasive wear in a humid and warm environment, which may explain the substantial discrepancies in the comparison of in vitro aging and the retrieved ZTA samples [95]. The underestimation of phase transformation might be critical in dentistry as well, where materials are constantly subjected to low-intensity loads and cumulative damage is produced in a moist/warm environment. Moreover, several factors should be considered in the evaluation of phase transformation in polycrystalline composites for dental applications, such as mechanical stress, surface reactions from porcelain veneers, and from grinding instruments. Therefore, it is clear that the LTD process of zirconia-based materials is significantly more complex than what was assumed in the previous literature and warrants further investigations considering the testing methods that include relevant factors for restorative use.

Considering compositions with higher content of zirconia, ATZ composites have been considered as excellent structural materials with increased fracture toughness in relation to ZTA composites [85]. In fact, an ATZ composed of tetragonal zirconia co-doped with yttria and niobium (Al_2_O_3_/Y[Nb]-TZP) was introduced in dentistry almost a decade ago for the manufacture of abutments for implant-supported prostheses and has demonstrated a high probability of survival (97%) in up to 7 years of follow-up [96]. 

While the aging kinetics were described as being slower in ATZ when compared to 3Y-TZP, the composite is still susceptible to LTD [97]. The reduced transformation (the XRD patterns of ATZ before and after aging is presented in Figure 8B) along with the presence of residual compressive stresses observed in the aged ATZ composites resulted in an increased flexural strength after aging, different to the first-generation 3Y-TZP, where a significant reduction in strength was observed with the same amount of phase transformation [82]. 

From a commercial perspective, the most promising ATZ reported in the literature to date, due to its high mechanical properties [98], is a nanosized composite with alumina particles dispersed in a cerium-stabilized tetragonal zirconia matrix (Ce-TZP/Al_2_O_3_, NANOZR developed by Panasonic). Furthermore, the most remarkable result in the current history of the development of a zirconia of high stability and strength was obtained in the “LONGLIFE European Project” (Advanced multifunctional zirconia ceramics for long-lasting implants, 7th European Framework Program), in which a multiphase material (84% Ce-TZP with 8 vol% Al_2_O_3_ and 8 vol% SrAl_12_O_19_) was developed. This material demonstrated high aging resistance, strength (1100 MPa), fracture toughness (>10 MPa√m), and an exceptionally high Weibull modulus (*m* = 60), the highest ever reported for a ceramic and close to that reported for metals [13,99]. 

## 3. Discussion and Clinical Considerations

Classification systems for dental ceramics are relevant for a variety of purposes, among them, to allow for standardization and clear communication in scientific reports and between professionals. While different systems have been proposed to classify dental ceramics, the focus on chemical composition as the guiding parameter has been considered as a logical step that easily allows for the inclusion of new restorative materials and provides useful information regarding materials’ properties and clinical indications [14]. With the development of zirconia-based materials, different classification systems have emerged, often based on “zirconia generations” determined by modifications in the composition (e.g., yttria content) that affect mechanical and optical properties. Therefore, different “generations of dental zirconia” have been proposed according to the significant improvements in translucency, which were achieved through modifications in the microstructure, composition, and processing [15,35,100]. 

While the classification of zirconia by generations is useful to understand their temporal evolution, their continued development will inevitably lead to a vast number of generations that, at some point, shall become confusing for scientists and clinicians. Therefore, the updated composition-based classification proposed in this review aims to simplify the understanding of novel developments in polycrystalline ceramics. First- and second-generation 3Y-TZP [15] have been unified in this paper and sub-grouped according to their differences in composition and sintering temperature as well as clinical indications as infrastructure and monolithic 3Y-TZP. When more than 4 mol% of yttrium oxide is used to stabilize the zirconia’s cubic phase, more translucent and materials with reduced toughness are obtained, which have been considered as the third and fourth generations of dental zirconias [35]. and were classified in this manuscript as belonging to the 4Y-PSZ and ≥5Y-PSZ families, respectively. While 4Y- and 5Y-PSZ have been considered as “ultra-translucent” zirconias, some differences in mechanical, optical, and aging behavior have been reported between both materials, with higher translucency, hydrothermal stability, and lower fracture toughness for formulations with a higher cubic phase content [39,101]. Finally, the development of novel zirconia compositions aiming to mimic the pigmentation and translucency of natural teeth led to the development of multichromatic and multilayered zirconias, respectively. 

**Figure 9:**
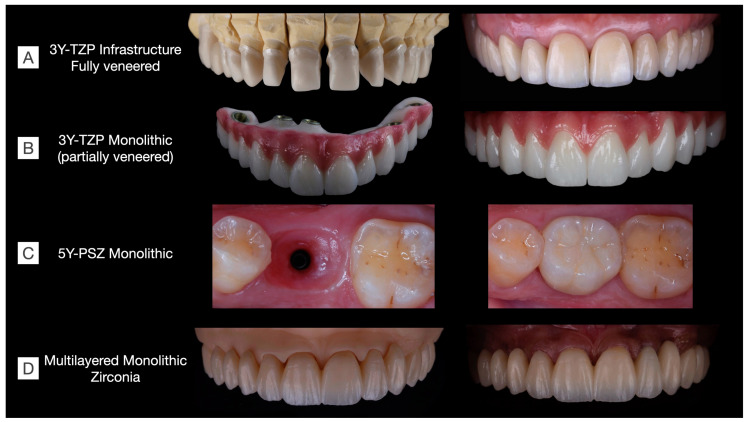
Clinical cases rehabilitated with different kinds of zirconias. (**A**) 3Y-TZP infrastructures fully veneered with feldespathic ceramic in a tooth-supported upper arch rehabilitation including single-crown and three-unit fixed dental prostheses (Clinical case conducted by Benalcázar-Jalkh EB, and Pegoraro LF, and Dental Technician M. Portaluppi). (**B**) Partially veneered monolithic zirconia used for full-arch implant-supported prosthesis. The buccal aspect of the teeth and gingiva were veneered to achieve esthetic results, while occlusal and palatal aspects were designed in monolithic zirconia to reduce the risk of porcelain chipping (Clinical case conducted by Laura Firmo de Carvalho and Dental Technician Marcos Celestrino). (**C**) 5Y-PSZ, or “ultra-translucent” zirconia, was used to manufacture an implant-supported single crown over a Ti-base abutment (Clinical case conducted by Raphaelle SM de Sousa and Dental Technician/DDS Ricardo Tanaka). (**D**) Full-arch tooth-supported prosthesis manufactured in a monolithic multilayered zirconia (3Y-TZP/5Y-PSZ) and stained to achieve esthetic results (Clinical case conducted by Benalcázar-Jalkh EB, Bonfante EA, and Dental Technician M. Celestrino).

As discussed in the previous sections, the trade-off between optical and mechanical properties is challenging in the development of zirconia materials. Considering clinical indications, there is currently no commercially available material that is strong, esthetic, and hydrothermally stable for the manufacture of long-span prostheses. In brief, the following issues have been temporally reported: (1) veneered 3Y-TZP present a high risk for porcelain chipping [3,9,27,55]; (2) monolithic 3Y-TZP is opaque and prone to low-temperature degradation [59], with no clinical studies to confirm its survival in the long term; (3) 4Y-PSZ is recommended for up to three-unit FDPs [35]; (4) 5Y-PSZ is not recommended for FDPs in the posterior area nor for long-span FDPs [15]; (5) multichromatic systems present the same concerns that monolithic 3Y-TZP and 4Y- and ≥5Y-PSZs; and (6) novel multilayered zirconias appear an interesting option with the combination of the advantageous properties of zirconias that have a gradient structure provided by the different amounts of stabilizers in each layer [45]. While esthetic outcomes can be achieved through the laboratorial staining of multilayered systems (Figure 9), clinical studies are warranted to evaluate their long-term clinical performance.

Oral rehabilitation necessitates the careful selection of restorative materials tailored to each clinical situation. In Figure 9, various clinical cases are depicted, each rehabilitated using different families of zirconias. Among the monolithic alternatives to fully veneered 3Y-TZP, the partially veneering of translucent 3Y-TZP has been proposed to mitigate the risk of porcelain chipping. However, concerns persist regarding the metastability of monolithic 3Y-TZP, particularly in unveneered and occlusal areas, where t-m phase transformation, along with surface crushing and grain pull-out due to tribological stress, are expected, though their long-term effects on prosthesis performance remain uncertain [58]. Furthermore, laboratorial-colored 5Y-PSZ and multilayered zirconia can be used to manufacture esthetic single-unit and long-span fixed dental prostheses, respectively. These materials have recently been introduced to the market, and as a result, there is currently a lack of long-term clinical evidence regarding their performance.

Although the improvement in translucency has driven the development of zirconia materials, it is clinically relevant to consider challenging scenarios, such as darkened substrates and titanium abutments, where translucent materials might compromise the outcome of the prosthetic treatment. In fact, in vitro research has suggested that monolithic zirconia with different yttria contents and milled lithium disilicate are able to adequately mask the normal dentin shade; however, none of them was capable of masking severely discolored dentin [102]. In such cases, an increased masking ability and enough material thickness are required to successfully hide darkened substrates.

## 4. Future Perspectives and Concluding Remarks

While the significant developments in polycrystalline ceramics in recent years have led to a broad diversity of materials with different compositions, properties, and clinical indications, the implementation of strong, esthetic, and sufficiently stable materials for long-span FDPs has not been completely achieved. Table 1 presents a summary of the type, clinical indication, and susceptibility to low-temperature degradation of polycrystalline ceramics for dental reconstructive applications. 

Although several improvements have been observed regarding the translucency of monolithic zirconias, LTD and the trade-off between mechanical and optical properties remain a concern. Furthermore, mechanical complications due to the fracture of the veneer porcelain in porcelain fused to zirconia and the lack of clinical data in the mid- and long term for monolithic zirconias represent an opportunity for further developments in materials science.

As for the steps in development and future perspectives, there are several aspects that must be taken into consideration regarding the search for resistant and stable materials. Among the routes for the development of strength and aging-resistant zirconias, grain boundary engineering [103,104] and the search for alternative zirconia-based systems are highlighted [105]. Other ways of stabilizing zirconia include the use of tetravalent cations or combinations of cations with compensating charges [106]. Furthermore, there is no doubt that the results achieved in the “LONGLIFE European Project” constitute a breakthrough in the synthesis of aging-resistant polycrystalline ceramics [13,99], in which the synthesis of ceria-stabilized zirconia composites containing equiaxed alumina and elongated strontium hexa-aluminate (Ce-TZP-Al_2_O_3_-SrAl_12_O_19_) resulted in polycrystalline ceramics with a high flexural strength (1100 MPa), fracture toughness (>10 MPa√m), and resistance to hydrothermal degradation. Furthermore, an outstanding Weibull modulus (m = 60) with almost no dispersion in strength data was reported for three-phasic ceria-stabilized zirconia composites, making them a suitable alternative to balance strength, toughness, and stability in humid environments [13,99].

Another approach presented by Prof. Chevalier’s group comprises the development of high-strength nanosized 1.5 and 3 mol% yttria-stabilized zirconia for dental applications, where decreasing the grain size below 100 nm was reported to be effective in the reduction in the scattering coefficient. The stabilization of tetragonal nano-grains with 3 mol% of Y_2_O_3_ resulted in outstanding mechanical properties (flexural strength of 1600 MPa; fracture toughness of 3.3 MPam^1/2^), aging resistance, and improved translucency in relation to submicrometric zirconia (3Y-, 4Y- and 5Y-TZPs) produced with commercially available powders [107,108]. However, the processing of nanosized yttria-stabilized zirconia is more complex and time-consuming than conventional powder processing of sub-micrometric-sized yttria-stabilized zirconia [107,109]. Apparently, this material is commercially available as dental implants, but are yet to be introduced into the market as prosthodontic restorative materials and further investigations to assess their performance for dental applications are warranted. 

Among the innovative approaches explored in the field of polycrystalline ceramics, the development of functionally graded materials is noteworthy [110]. Materials with functional gradients present discrete or continuous variation in composition, structure, and properties along the structure, with the objective of presenting superior properties regarding the material with a homogeneous composition. This process has been incorporated into both zirconia and alumina after pre-sintering, where through the infiltration of a glass with a compatible coefficient of thermal expansion, both materials benefited from an increase in mechanical properties. Additionally, the glass layer in the inner surface allows for conventional adhesive cementation and, on the external surface, improves the esthetic appearance [111,112].

Considering that milling processes are well established and have been routine for many years, it is indisputable that they will remain for some time as the main method for prosthetic manufacture. However, the excessive waste of material, the environmental impact, and the wear of the CAM drills have directed attention to the additive manufacturing (AM) of dental ceramics for several years [113]. Among some of the primary challenges of the additive manufacturing of ceramic restorative materials, some concerns, such as surface quality, dimensional accuracy, and mechanical properties that are directly influenced by defects introduced in ceramic parts during AM, still need to be overcome [114]. Significant advances, however, have been observed in recent years, including the manufacturing of long-span monolithic zirconia prostheses with marginal adaptation superior to their milled counterparts, and with details of occlusal anatomy currently not reproduced by milling [115]. 

Despite the advances reported for the AM of zirconia materials, the high heterogeneity and low certainty of evidence regarding mechanical properties and accuracy of 3D-printed dental zirconias for restorative purposes [116] warrant further investigation before clinical application. Furthermore, the interest in the additive manufacturing of dense polycrystalline ceramics for industrial and biomedical applications are in line with sustainable requirements, promoting waste reduction and opening possibilities for the use of recycled materials [117,118]. While a handful of studies have addressed such a possibility, there is a clear struggle to achieve favorable mechanical properties using recycled zirconia for 3D-printed dental prostheses [119], where a 37% decrease in flexural strength has been reported for 3D printing with recycled zirconia (389 MPa) regarding the same process with pristine zirconia (1057 MPa) [119]. Such problem has been previously reported in a different experimental set-up for recycled pressed zirconia, where pristine and recycled powders were mixed in concentrations ranging from 5% to 50% of recycled material [120]. The recycled powder characterization evidenced significant morphological differences compared to its pristine counterpart, which explains the significant reduction in density and flexural strength observed as the recycled powder content increases [120]. While this recycling route may allow for the re-utilization of zirconia for applications such as jewelry or processing of refractories, further processing and different recycling methods are required to allow for its reusage for dental applications, where higher mechanical properties and reliability are required. Therefore, significant efforts in research and development along with large investments in materials and technology for the AM of recycled and pristine dense polycrystalline ceramics should be expected in subsequent years.

## Figures and Tables

**Table 1:** Summary of the type, clinical indication, and susceptibility to low-temperature degradation of polycrystalline ceramics.

		Clinical Indication					
	Framework (F) Monolithic (M)	Veneer	Partial Coverage Restoration	Full Crown Anterior (A) Posterior (P)	FDP	Implant Abutment	LTD
Polycrystalline Ceramics							
Zirconias							
Framework 3Y-TZP	F	-	-	✓(A/P)	✓	✓	Y
Monolithic 3Y-TZP	M ^	-	✓	✓ (A/P)	✓	✓	Y
4Y-PSZ	M	-	✓	✓ (A/P)	✓ •	✓	Y
≥5Y-PSZ	M	✓	✓	✓ (A/P)	✓ •	-	N
Multichromatic	M	-	✓	✓ (A/P)	✓ *	-	Y *
Multilayered	M	-	✓	✓ (A/P)	✓ *	-	Y *
Polycrystalline composites (ZTA and ATZ)	F	-	-	✓ (A/P)	✓	✓	N

FDP: Fixed dental prostheses; LTD: Low-temperature degradation; Y: Yes; N: No. * Multichromatic and multilayered indication for FDPs (implant and tooth-supported) and LTD susceptibility depends on its composition. It is always recommended to follow the manufacturers’ instructions. ^ Monolithic zirconia, especially 3Y-TZP, may be partially veneered with porcelain for improved esthetics. • 4Y-PSZ is recommended by most manufacturers for up to three-unit FDPs in the anterior and posterior regions. 5Y-PSZ is recommended for three-unit FDPs in the anterior region or as premolar pontic. It is advised to check the specific material’s instructions for clinical indications.

## Data Availability

Data will be provided by the authors upon request.
